# A Systematic Review and Comparative Analysis of Botox Treatment in Aesthetic and Therapeutic Applications: Advantages, Disadvantages, and Patient Outcomes

**DOI:** 10.7759/cureus.67961

**Published:** 2024-08-27

**Authors:** Christopher R Meretsky, Joseph P Umali, Anthony T Schiuma

**Affiliations:** 1 Surgery, St. George's University School of Medicine, Great River, USA; 2 College of Pharmacy, St. John's University, Queens, USA; 3 Medicine, St. George's University School of Medicine, Great River, USA; 4 Orthopedic Surgery, Holy Cross Hospital, Fort Lauderdale, USA

**Keywords:** safety and efficacy, patient outcomes, therapeutic applications, aesthetic applications, botox

## Abstract

The objective of this review article is to comprehensively analyze Botox treatment, emphasizing its aesthetic and therapeutic applications, along with the associated advantages, disadvantages, and patient outcomes. By reviewing current literature, the article evaluates Botox's efficacy and safety in various clinical settings, including cosmetic procedures and medical treatments for conditions such as chronic migraines and excessive sweating, while also exploring patient satisfaction and potential risks. This review article provides a comprehensive analysis of Botox treatment, focusing on its aesthetic and therapeutic applications, as well as the associated advantages, disadvantages, and patient outcomes. Botulinum toxin, derived from *Clostridium botulinum*, is widely recognized for its ability to induce temporary muscle paralysis, leading to significant improvements in both cosmetic and medical conditions. The article reviews findings from recent clinical trials, case reports, and observational studies, highlighting the efficacy and safety of Botox in treating various conditions such as chronic migraines, excessive sweating, and muscle spasticity, alongside its cosmetic use in wrinkle reduction. While the benefits of Botox are substantial, including its minimally invasive nature and high patient satisfaction rates, potential risks and complications, including rare adverse events, are also discussed.

## Introduction and background

Botulinum toxin, commonly known as Botox® (Allergan Inc., Irvine, CA, USA), is a protease exotoxin derived from Clostridium botulinum [1]. Botulinum neurotoxins are generated by various strains of bacteria belonging to the Clostridium genus, especially Clostridium botulinum. There are seven recognized serotypes of botulinum neurotoxin (A-G), with types A (BTA) and B (BTB) being the ones commercially produced for medical applications. The botulinum toxin is initially synthesized as a 150-kDa protein, which undergoes posttranslational modifications to form a 100-kDa heavy chain and a 50-kDa light chain, connected by a disulfide bond [2]. Botulinum neurotoxins induce flaccid paralysis of targeted muscles by inhibiting the release of acetylcholine (ACh) from vesicles at the neuromuscular junction by inhibiting presynaptic exocytosis at cholinergic nerve endings in the peripheral nervous system, resulting in the paralysis of skeletal muscles. This leads to paralysis and a significant reduction in motor end plate potentials within just a few hours following the injection of the neurotoxin [3]. It functions by inhibiting the release of acetylcholine from cholinergic nerve endings, resulting in reduced activity of muscles or glands. The effects of Botox are temporary and can be adjusted by altering the dosage and frequency of administration [4]. The usual duration of botulinum toxin's effects ranges from three to four months, influenced by various factors such as dosage, concentration, injection method, patient immune response, and more. In the preapproval studies for each commercially available product, the glabellar area was treated, and the number of patients sustaining a response was monitored on a monthly basis [5].

Known for being one of the most powerful naturally occurring biological toxins, Botox was responsible for numerous accidental fatalities before it was utilized in the medical field. Its first therapeutic application was in 1980 for treating strabismus. In 1989, the toxin's cosmetic effects on wrinkles were observed, but it wasn't until 2002, following approval from the Food and Drug Administration (FDA), that Botox became widely popular as a non-surgical cosmetic alternative [6].

The objective of this review article is to provide a comprehensive analysis of Botox treatment, focusing on its aesthetic and therapeutic applications, as well as the associated advantages, disadvantages, and patient outcomes. By synthesizing current literature, the article aims to evaluate the efficacy and safety of Botox in various clinical settings, including cosmetic procedures and medical treatments for conditions such as chronic migraines, excessive sweating, and muscle spasticity. Additionally, the review will explore patient satisfaction and quality of life improvements resulting from Botox interventions, while also addressing potential risks and complications.

## Review

Methods

**Figure 1:**
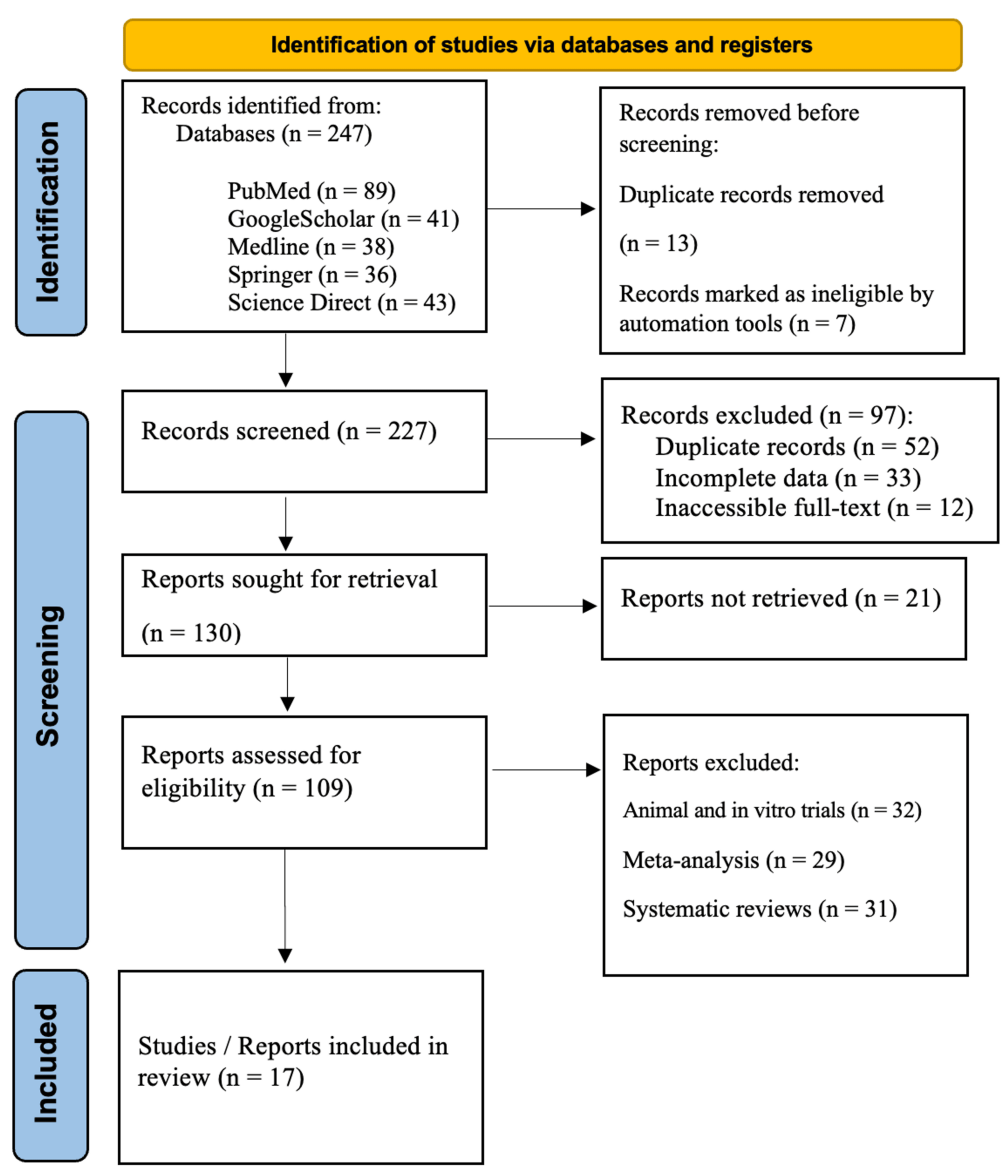
PRISMA flowchart: literature search and study selection n, number; PRISMA, Preferred Reporting Items for Systematic Reviews and Meta-Analyses [7]

In the current review, we conducted a comprehensive literature search utilizing various scholarly databases, including PubMed, Google Scholar, Medline, Springer, and ScienceDirect. To ensure a thorough exploration of the topic, we employed a variety of relevant keywords such as “Botulinum toxin/Botox Treatment,” “Botulinum toxin/Botox in Aesthetic Applications,” “Botulinum toxin/Botox in Therapeutic Use,” “Advantages of Botox Treatment,” and “Disadvantages of Botox Treatment.” This systematic approach allowed us to obtain a well-rounded understanding of the subject, facilitating an in-depth analysis of both the benefits and drawbacks associated with Botox treatment in both aesthetic and therapeutic contexts. Through this literature review, we aim to provide valuable insights that contribute to the existing body of knowledge on Botox and its implications in medical practice. The Preferred Reporting Items for Systematic Reviews and Meta-Analyses (PRISMA) guidelines were followed to ensure transparency and reproducibility in the review process (Figure 1).

Study Selection Criteria and Process

Inclusion criteria: The criteria for selecting studies for our review were as follows: We included studies published between 2014 and 2024 that reported on the outcomes of therapeutic and aesthetic applications of botulinum toxin/Botox. It was essential that these studies involved clinical trials.

Exclusion criteria: On the other hand, we excluded certain studies from our selection. These included studies that did not provide sufficient data on therapeutic and aesthetic applications of Botox. We also excluded meta-analysis studies, reviews, and editorials that did not contain original data. Lastly, studies that were conducted exclusively on animals were not considered for our research. This careful selection process ensured the relevance and reliability of our review.

Results

Botox in Therapeutic Applications

Table 1 summarizes the clinical trials, case reports, and observational studies on the therapeutic uses of Botox over the past 10 years (2014-2024). The findings indicate several advantages of this therapeutic toxin. Botox is a first-line treatment for spasmodic dysphonia and an effective solution for both medical and dental applications. In addition, the use of Botox in dentistry has risen, as it appears to be a successful treatment for excessive saliva production, with limited invasiveness and minimal risks. Botox is more effective at reducing pain in anal fissures and levator ani syndrome. Furthermore, a single injection of botulinum toxin type A (BTXA) effectively alleviates knee pain and enhances the quality of life for older adults with knee osteoarthritis. Moreover, Botox considerably decreases the frequency of headaches and migraines while improving the quality of life for individuals with chronic migraine. Additionally, Botox is a safe and effective treatment for hemifacial spasm, and Botox injections demonstrate a 77% effectiveness in alleviating chronic temporomandibular disorders, with a lasting effect, especially in children with esotropia.

**Table 1:** The last 10 years (2014-2024) of clinical trials, case reports and observational studies on the therapeutic applications of Botox

Reference	Condition treated	Study design	Objective	Conclusion
Hirose K et al. [8]	Spasmodic dysphonia (SD)	Placebo-controlled, randomized, double-blinded parallel-group comparison/open-label clinical trial	Analyze the detailed chronological course and clinical factors that affect the therapeutic effect of BT (Botox) for SD	Botox is a first-line treatment for spasmodic dysphonia, but the detailed chronological course and clinical factors affecting the therapeutic effect have been vague. This study aimed to clarify these aspects.
Speles E et al. [9]	Bruxism	Placebo-controlled, randomized clinical trial	Evaluating the therapeutic effect of Botox on patients with bruxism	Botox is an effective solution for both medical and dental applications. BTXA addresses the primary concerns of bruxism, including excessive tooth grinding and muscle tension.
Agyei JO et al. [10]	Cervical kyphotic deformity	A case report	Looking at an older woman who experienced intense neck pain following a car accident. She received Botox injections in her neck muscles, leading to a significant cervical kyphotic deformity.	This situation shows a risk that can come from giving Botox in the neck of an older patient with possible neck issues like weakness, instability, or curvature. It highlights the importance of checking for these problems before deciding to use Botox on patients who could end up with neck deformities.
Alvarenga A et al. [11]	Salivary glands for drooling	Observational study	Evaluate the results of using BOTOX-A injections in children with excessive drooling.	BOTOX-A appears to be a successful treatment for excessive saliva production that involves limited invasiveness and minimal risks.
Bibi S et al. [12]	Anal pain	Observational study	Assessing the effectiveness of Botulinum toxin-A (Botox) in alleviating anal pain related to anal fissures (AF) and Levator ani syndrome (LS).	Botox is more effective at reducing pain in AF than in LS. It is a good choice for LS cases that do not respond to medical treatment. Using higher amounts of Botox in LS is acceptable, but should be monitored. Botox injections tend to have few complications.
Najafi S et al. [13]	Knee osteoarthritis	Clinical trial	To evaluate the effectiveness of a single injection of abobotulinumtoxin A into the knee joint cavity for pain relief in older adults.	A single injection of botulinum toxin type A effectively alleviates knee pain and enhances the quality of life for older adults with knee osteoarthritis.
Khalil M et al. [14]	Chronic migraine	Observational Study	To assess the efficacy and safety of BOTOX in adults with chronic migraine	BOTOX considerably decreases the frequency of headaches and migraines while enhancing the quality of life for individuals with chronic migraine. However, its expense may restrict its adoption as a primary treatment option.
Lai KKH et al. [15]	Hemifacial spasm	Observational Study	The objective of this report is to evaluate the therapeutic outcomes of Botox and eyelid surgery in individuals diagnosed with hemifacial spasm (HFS).	Botox is a safe and effective treatment for HFS. For patients with coexisting eyelid conditions, eyelid surgery can enhance the effectiveness of subsequent Botox injections by increasing patient satisfaction and lowering the rate of complications.
Connelly ST et al. [16]	Chronic temporomandibular disorders (TMD)	Observational Study	Evaluating the clinical outcomes associated with the administration of Botox injections in alleviating the symptoms of chronic temporomandibular disorders (TMD).	Botox injections demonstrate a 77% effectiveness in alleviating chronic temporomandibular disorders, particularly showing greater improvement in patients with bruxism and stress-related psychiatric comorbidities.
Wipf M et al. [17]	Misalignment of the Eyes	Observational Study	To assess the therapeutic use of Botox to correct misalignment of the eyes	The treatment has a lasting effect, especially in children with esotropia. It is a minimally invasive procedure and can help prevent the need for surgery.

While disadvantages of using Botox in therapeutic applications were rare, one study did demonstrate a potential risk of giving Botox in the neck of an older patient with possible neck issues, such as weakness, instability, or curvature.

Botox in Cosmetic Applications

Table 2 summarizes the findings from the last decade's clinical trials, case reports, and observational studies on the aesthetic applications of Botox. These results highlight both the benefits and drawbacks of Botox treatment as evidenced by human studies. Among the notable advantages are: onabotulinumtoxin A (ONA, Allergan Inc.) and NBT (NABOTA®, Daewoong Pharmaceutical, Seoul, Korea) demonstrate similar efficacy and safety profiles in reducing masseter size; the use of Botox in dentistry has experienced significant growth; the multipoint and multilevel injection technique (MMIT) effectively minimizes facial wrinkles while maintaining a natural appearance and offering personalized aesthetic outcomes; Botox provides a less invasive, quicker, and safer alternative for aesthetic enhancement; when administered to the appropriate muscles at the correct dosages, Botox can achieve harmonious and visually appealing results that complement an individual's smile; and the application of Botox for orofacial pain has been associated with improvements in facial aesthetics. Conversely, it is important to note that only one study reported a negative outcome, highlighting a rare instance of sudden death following a Botox injection.

**Table 2:** The last 10 years (2014-2024) of clinical trials, case reports and observational studies on the aesthetic applications of Botox

Reference	Study design	Subjects and condition	Objective	Parameters considered	Main outcomes	Conclusion
Yoelin SG et al. [18]	Randomized controlled trial	42 subjects who received EB-001 or placebo	Assessing the safety and effectiveness of EB-001, a new type of Botox, in individuals with glabellar frown lines.	Severity of glabellar frown lines; Adverse events, physical and examinations laboratory tests.	The incidence of adverse events was low, with mild to moderate headaches identified as the most common occurrence; No serious adverse events or ptosis were reported, and there were no clinically significant changes in other safety assessments;	EB-001 showed favorable safety, tolerability, and dose-dependent efficacy, with an 80 percent response rate at the highest dose. The maximum clinical effect of EB-001 was seen within 24 hours and lasted between 14 and 30 days.
Wanitphakdeedecha R et al. [19]	Randomized controlled trial	35 subjects diagnosed with bilateral symmetrical masseter hypertrophy through physical palpation	To compare the efficacy and safety of Onabotulinumtoxin A (ONA) and Botulinum toxin type A and NBT for masseter muscle reduction.	Standardized photographic; The mean volume of the masseter muscle; Patients' satisfaction levels and any side effects.	There was no statistically significant difference in the average volume between the two sides; The mean masseter volume at 3- and 6-month follow up visits reduced significantly on both ONA and NBT sides; No side effect reported on both sides after injection.	ONA and NBT offered similar efficacy and safety in reducing masseter size.
Demyati AK et al. [20]	Observational Study	255 patients	To examine the participants' viewpoints on the role of Botox in dentistry.		Participants' awareness of several Botox indications, including wrinkle reduction, treatment of muscle spasms, and management of a gummy smile; Participants identified infections, bruising, and eyelid drooping.	The use of Botox in dentistry has seen significant growth. While participants demonstrated a satisfactory understanding of Botox in aesthetic practices, they were less informed about its aesthetic applications.
Iozzo I et al. [21]	Observational Study	223 patients treated for facial wrinkles utilizing a novel approach known as the multipoint and multilevel injection technique (MMIT) for the administration of botulinum toxin A (BTA).	Patient satisfaction was evaluated using a standardized Facial Line Treatment Satisfaction Questionnaire (FTSQ), which comprises a series of predefined questions presented in a fixed and consistent sequence.		In individuals with youthful skin, facial wrinkles were completely eliminated, while in those with older skin, the wrinkles were significantly diminished, resulting in a natural appearance for both static and dynamic expressions; The application of botulinum toxin by MMIT allows for more precise control, resulting in softer and more natural-looking outcomes.	The multipoint and multilevel injection technique (MMIT) successfully minimizes facial wrinkles while ensuring a natural look, delivering personalized aesthetic results.
Pedron IG et al. [22]	Case Report	A patient who presented dentogingival discrepancy and gummy smile	Discussing a patient who exhibited a dentogingival discrepancy and a gummy smile.		The use of Botox offers a less invasive, quicker, safer, and more effective alternative. When applied to the appropriate muscles with the correct dosage, it yields harmonious and aesthetically pleasing results in relation to the desired smile.	This technique serves as a valuable complementary approach for enhancing the aesthetics of the smile and achieves improved outcomes when used alongside resective gingival surgery.
dos Santos AP et al. [23]	Case Report	A patient who presented with a headache accompanied by pain in the glabellar and frontal areas.	To present the case of a patient who experienced a headache along with pain in the glabellar and frontal regions.	Adverse events such as pain at the injection site, hematomas, infection, edema, eyelid ptosis, and asymmetry	The use of Botox for orofacial pain is linked to facial aesthetic improvements. These applications, performed by dental surgeons, can enhance patients' self-esteem and quality of life, alongside their anticipated therapeutic benefits.	Botox has emerged as an effective therapeutic and aesthetic option for various dental applications.
Mya N et al. [24]	Case report	A woman collapsed at a beauty salon shortly after receiving a Botox injection.			Despite resuscitation efforts in the hospital's emergency unit, she was unable to be revived and unfortunately passed away.	This case report emphasizes the rare occurrence of sudden death following a Botox injection. Researchers encourage individuals to be aware of the potential unforeseen risks associated with cosmetic procedures while seeking beauty enhancements.

Discussion

Botox in Therapeutic Applications

Advantages: The findings of the present review show that Botox has demonstrated several therapeutic benefits, including being a first-line treatment for spasmodic dysphonia, an effective solution for both medical and dental applications, and successful in reducing pain associated with conditions like anal fissures, levator ani syndrome, and knee osteoarthritis. Botox has also been shown to considerably decrease the frequency of headaches and migraines, as well as being a safe and effective treatment for hemifacial spasm and chronic temporomandibular disorders. While the use of Botox in therapeutic applications is generally safe, one study did identify a potential risk of administering Botox in the neck of older patients with certain neck issues. Overall, the findings from the past 10 years of clinical studies support the expanding therapeutic uses of Botox.

In a recent study conducted in 2022, researchers assessed the effectiveness of botulinum toxin injections both as a standalone treatment and in combination with Profhilo gel for neck rejuvenation. Patients who sought neck rejuvenation and met the inclusion criteria were randomly assigned to one of two groups. Authors of this randomized clinical trial concluded that combination of Dysport Botox and Profhilo gel offers a safer and more effective solution for addressing neck aging in patients who are not candidates for surgery, compared to using Dysport Botox alone [25]. In the same background, Mierzwa et al. (2022) indicated that Botox injections reduce stiffness in the masticatory muscles, specifically the masseter muscle. This, however, can paradoxically increase the stiffness of the temporalis muscle. This is likely due to a compensatory mechanism in response to the altered function of the masseter. As a result, simultaneous evaluation and treatment of the temporalis muscle may be required alongside Botox injections to the masseter to achieve the desired functional and cosmetic outcomes [26].

Botox has proven to be highly effective in treating various non-cosmetic conditions related to Otorhinolaryngology and Head & Neck Surgery. As research continues to expand, both the scope of clinical applications and the number of patients receiving Botox are likely to grow. It truly lives up to its reputation as "the poison that heals" [27].

Botox can serve as a valuable therapy for various pain management scenarios where other treatments may not yield results. It is also beneficial for acute pain, though its use should be limited. For instance, administering a pre-operative injection of BTXA has demonstrated the ability to alleviate and manage post-operative pain effectively. The well-established antinociceptive effects of BTXA are mainly due to its ability to inhibit the release of neurotransmitters and neuropeptides, as well as its interference with the fusion process in neuronal cells. However, the presence of cleaved SNAP-25 at locations distant from the administration site suggests a potential for a combined action. Botulinum neurotoxins' fusion proteins that incorporate neuropeptides, like SP-LC, show promise as innovative new treatments for pain management. Additionally, Botulinum neurotoxins' impact on non-neuronal cells may provide valuable insights into the mechanisms behind its effectiveness in alleviating pain symptoms [28]. Botulinum neurotoxin therapy has proven to be a valuable alternative to traditional medical treatments for various conditions affecting the head and neck, particularly regarding morbidity, mortality, and patient satisfaction with treatment results. Botulinum neurotoxin therapy offers effective alternatives to conventional treatment methods for certain neurological conditions affecting the head and neck region. This therapy can help alleviate some of the complications associated with standard treatments. However, further research is necessary to establish the optimal dosages of botulinum neurotoxin for treating various conditions that impact the head and neck areas [29].

A recent study reported that the incorporation of Botox into the diagnostic and therapeutic process for chronic migraine within an Italian Local Health Unit has demonstrated positive impacts on clinical outcomes and resource allocation for the Italian National Health Service. By strategically integrating this treatment into the patient care pathway, healthcare providers can optimize the management of chronic migraine while ensuring efficient utilization of available resources [30]. Similarly, a clinical trial study shows that incorporating Botox into the comprehensive management of chronic migraine, alongside other evidence-based interventions such as preventive medications and lifestyle modifications, can optimize patient outcomes and reduce the socioeconomic impact of this condition [31].

Bruxism can be managed effectively through local intramuscular injections of Botox type A, administered two to four times a year. This approach addresses the underlying cause of the condition, unlike conservative treatments such as nocturnal splints, which primarily prevent the complications associated with teeth grinding. Botox serves as an effective solution from both medical and dental perspectives, as it alleviates the primary issues related to bruxism, including excessive occlusion and muscle tension [32]. Recent reports indicate that Botox injections are becoming a promising therapeutic option, particularly for patients who struggle with compliance or haven't experienced symptom relief from conventional treatments. This option remains notable despite its high cost and potential for temporary discomfort [33].

The evolution of Botox from a toxic substance to an incredibly versatile therapeutic tool has expanded the possibilities in dentistry. It has proven to be particularly beneficial for managing cases that do not respond to less invasive treatments or when used alongside them. Botox provides a minimally invasive option for addressing specific cases, resulting in fewer complications. Nonetheless, it is essential for practicing dentists to ensure that such treatments fall within their professional scope and that they possess the necessary training to administer Botox safely and handle any potential adverse effects [34].

Disadvantages: According to data of the present literature, although the disadvantages of using Botox in therapeutic applications are generally uncommon, one study highlighted a potential risk associated with administering Botox in the neck of older patients with pre-existing neck issues, such as weakness, instability, or curvature. In this case, the injection exacerbated the patient's underlying problems, raising concerns about the suitability of Botox treatment in sensitive anatomical regions. The findings suggest that healthcare providers should exercise caution when considering Botox for older adults with known neck conditions, as the treatment could lead to complications. This emphasizes the importance of thorough patient assessments and careful consideration of individual medical histories before proceeding with such interventions.

In a recent case report, a 30-year-old woman was brought to our hospital showing signs like droopy eyelids, double vision, slurred speech, swallowing difficulties, and muscle weakness. She did not have any past illnesses. To enhance her appearance, she had received three Botox injections earlier. When examined physically, weakness in muscles like neck, eye, and throat was noted, leading to a diagnosis of myasthenia gravis. The signs of myasthenia gravis and overall impact of Botox are alike. When gathering medical history, this similarity must be noted. It is risky to inject Botox in large quantities (over 200 units per injection) or to administer frequent boosters within a short span. When systemic effects occur, treatment with therapeutic plasma exchange (TPE) can help reduce the circulating Botox levels [35]. In addition, a recent clinical trial study conducted in 2022 found that injecting 10 million units (MU) of BTXA into the masseter muscle reduced muscular activity in this muscle. This resulted in decreased muscle spasms and pain symptoms associated with nocturnal bruxism (teeth grinding during sleep) for approximately three months. However, the symptoms gradually returned as the effects of the BTXA injection wore off over time [36].

A recent study was designed to evaluate Dysport and Botox at a 2.5:1 ratio in the treatment of cervical dystonia (CD). A randomized, double-blind, multicenter, non-inferiority crossover study was conducted over at least 18 months. The results indicate that Dysport and Botox are similarly effective and safe for managing cervical dystonia at the specified ratio, although Botox demonstrated marginally superior outcomes [37].

The Aesthetic Applications of Botox

According to the outcomes of the current review, findings from the past decade regarding the aesthetic applications of Botox, based on clinical trials, case reports, and observational studies. Key advantages include comparable efficacy and safety of Onabotulinumtoxin A and NBT in reducing masseter size, the growing use of Botox in dentistry, and the effectiveness of the MMIT in minimizing wrinkles while achieving natural-looking results. Botox is recognized as a less invasive and quicker alternative for aesthetic enhancements and can yield visually appealing outcomes when used appropriately. However, it is important to acknowledge a rare negative outcome reported in one study, which noted a case of sudden death following a Botox injection.

Advantages: Botox has gained immense popularity as a cosmetic treatment due to its effectiveness in smoothing fine lines and wrinkles, resulting in a more youthful and refreshed appearance. By temporarily paralyzing the underlying facial muscles, Botox prevents the formation of dynamic wrinkles caused by repetitive facial expressions, leading to a rejuvenated look that can take years off one’s appearance. The treatment is minimally invasive, requiring little to no downtime after the quick injection process, which is a significant advantage for many patients. Additionally, Botox offers natural-looking results that can be tailored to address specific areas of concern without drastically altering facial expressions. When administered by a qualified and experienced provider, Botox proves to be a safe and effective option for achieving a smoother, more radiant complexion.

Sepehr et al. reported via an observational study that Botox injections for facial rejuvenation have a strong history of patient satisfaction, with consistent popularity in traditional injection sites and a growing trend in newer areas such as the superolateral orbicularis oculi and depressor anguli oris [38]. With the rise in the adoption of therapeutic techniques in aesthetic medicine, the incidence of complications stemming from various skin laser treatments, notably burns, has surged. Despite the scarcity of data and understanding surrounding the management of post-laser skin burns, a recent study by Soika (2022) has introduced an innovative strategy utilizing BTXA. This approach aims not only to provide pain relief and alleviate symptoms but also to diminish erythema and flushing resulting from the neovascularization of healing skin [39]. In addition, it was reported that the application of BTXA to relax depressor muscles prior to the injection of dermal fillers marks a significant shift in facial aesthetics. This strategy optimizes the advantages of both procedures, leading to improved long-term outcomes for patients, enhanced safety during the procedure, and greater overall satisfaction. As the aesthetics landscape advances, additional research and clinical trials are needed to strengthen the scientific foundation of this method and fine-tune its techniques [40].

In a recent study utilizing the FACE-Q to evaluate patient satisfaction after Botox type A treatment for glabellar rhytids, it was found that patient satisfaction with their overall facial appearance rose by 28% following the procedure. Participants indicated that they perceived themselves as looking approximately 5.6 years younger after treatment. Additionally, no significant differences were observed among the various treatment groups [41].

Disadvantages: While Botox can effectively diminish the appearance of wrinkles and fine lines, it also presents several potential drawbacks. The mechanism by which Botox operates involves temporarily paralyzing the underlying facial muscles, which can result in a "frozen" or unnatural appearance if not administered correctly. There is also a risk of the Botox migrating to unintended muscles, leading to uneven or asymmetrical results. Additionally, the effects of Botox are temporary, typically lasting only three to four months, necessitating repeat treatments to maintain the desired aesthetic. Patients may experience side effects such as headaches, bruising, or eyelid drooping. Long-term use of Botox raises concerns about a potential decrease in facial expressiveness over time. Furthermore, the cost of treatments can be significant, and some individuals may find the injections uncomfortable. Therefore, while Botox can be a valuable cosmetic tool, it is essential to carefully consider these potential disadvantages when contemplating aesthetic treatments.

In 2021, the FDA Adverse Event Report System was accessed using a web-based tool to identify the top 15 adverse events associated with four Botox brand names: Botox/Botox Cosmetic, Dysport, and Xeomin. Proportional reporting ratios (PRR) and relative odds ratios (ROR) were calculated for the analysis. Additionally, a literature review was conducted on eight clinically significant adverse events: eyelid/eyebrow ptosis, asthenia, muscular weakness, facial paresis, dysphagia, botulism, and death. The findings revealed that Botox/Botox Cosmetic had 38,367 adverse events reported, followed by Dysport with 3,582 events and Xeomin with 1,405 events. All brands that reported cases of eyelid and eyebrow ptosis demonstrated significant PRR and ROR values. However, the PRR and ROR values for asthenia were not significant across any of the drugs, while Dysport registered significant values for muscular weakness and dysphagia. Both Botox/Botox Cosmetic and Dysport showed elevated PRR and ROR values for facial paresis and botulism. Although there were reported cases of death related to Botox injections for all the brands, none of the PRR or ROR values for these cases were significant. Common adverse events linked to Botox injections include eyelid/brow ptosis and muscular weakness. Rare but concerning complications include dysphagia, botulism, and potentially death, due to the systemic spread of the toxin. This study represents the first analysis of adverse event data related to Botox injection reported to the FDA [42]. Similarly, Lee et al. (2020) indicated that numerous purported complications have been submitted to the FDA, many of which may not have resulted from injection or the direct effects of the toxin. Patients may have misattributed certain symptoms to BTXA, and healthcare providers should be ready to clarify any misconceptions. Common local complications included pain, swelling, and ptosis, which were generally not linked to severe patient outcomes. Nevertheless, constitutional symptoms require a more cautious approach [43].

## Conclusions

In conclusion, Botox treatment offers a range of aesthetic and therapeutic applications that come with both advantages and disadvantages. The benefits include effective reduction of wrinkles, facial rejuvenation, and various therapeutic uses for conditions such as chronic pain and excessive sweating, making it a versatile option for many patients. However, it is essential for practitioners and patients to be aware of potential risks, including adverse effects and rare but serious complications. Ultimately, a careful assessment of individual patient needs, expert administration, and informed consent are crucial in optimizing patient outcomes and ensuring a safe and effective experience with Botox treatments. As research continues to evolve, understanding these dynamics will enhance the overall quality and safety of Botox applications in both aesthetic and therapeutic contexts.
